# Delay for Tuberculosis Treatment and Its Predictors among Adult Tuberculosis Patients at Debremarkos Town Public Health Facilities, North West Ethiopia

**DOI:** 10.1155/2020/1901890

**Published:** 2020-09-19

**Authors:** Yibeltal Estemech Ayalew, Fikadu Ambaw Yehualashet, Worknesh Akanaw Bogale, Mengistu Berhanu Gobeza

**Affiliations:** ^1^Department of Medical Nursing, University of Gondar, Gondar, Ethiopia; ^2^Department of Community Health Nursing, University of Gondar, Gondar, Ethiopia; ^3^Department of Pediatrics and Child Health Nursing, University of Gondar, Gondar, Ethiopia

## Abstract

**Background:**

Delay in the diagnosis and treatment of tuberculosis exacerbates the disease and clinical outcomes. It further enhances transmission of the infection in the society as well as increased the severity of the illness and raised rate of mortality.

**Objectives:**

The major goal of this study is to determine the magnitude of delays in tuberculosis treatment and factors affecting tuberculosis treatment among adult tuberculosis patients at Debremarkos town, North West Ethiopia, 2018.

**Methods:**

Institution-based cross-sectional study design was employed. Systematically selected 300 adult TB patients were recruited to the study. The study was conducted at Debremarkos town public health facilities from March 1 to April 30, 2018. Logistic regression models were fitted to identify the predicting variables and control confounder's of the outcome variables. *P* value ≤ 0.05 with 95% CI was considered as an indicator for the presence of statistically significant association. The result revealed that the median total delay was 23 days (IQR: 19-28 days). The median patient and health system delays were 20 days (IQR: 15-20 days) and 4 days (IQR: 3-5 days), respectively. Tuberculosis patients living in a rural area were 1.14 times more likely to delay for the TB treatment (AOR: 1.141, 95% CI (1.106, 2.608)). Patients who were unable to read and write have almost two times a chance of being delayed (AOR: 2.350, 95% CI (1.630, 2.608)). Monthly income of patients has found another predictor for delay; patients with low monthly income were about six times more likely to delay for TB treatment (AOR: 6.375, 95% CI: (1.733, 23.440)). Those TB patients who had visiting traditional healers before arrival to health facilities were about 2.7 times more likely to delay for TB treatment(AOR: 2.795, 95% CI (1.898, 8.693)). *Conclusion and Recommendation*. The significant proportion of delays in tuberculosis treatment was found in this study. Living in the rural area, unable to read and write, lower monthly income, and visiting traditional healers were found independent predictors of TB treatment delay. The regional and zonal health administrator shall design various awareness creation mechanisms to educate the public about timely initiation of tuberculosis treatment.

## 1. Introduction

Tuberculosis (TB) is the ninth leading cause of mortality worldwide and the leading cause from a single infectious agent, ranking above HIV/AIDS [1]. Almost one-third of the world populations (about 2 billion) are infected with mycobacterium tuberculosis [2].

Treatment delay leads to more serious illness; as a result, it increased infectivity within the community and increased transmission of TB [3, 4]. Delay in initiation of treatment gratuitously prolongs the period of TB transmission within the community [5]. Delay in the diagnosis and treatment of tuberculosis results in increasing severity of the disease, chance of death, and risk of transmission of the infection in the society particularly among people who have contact with the patient. It is evident that, as delay for TB treatment progress, patients would become more contagious [6]. Delay in TB detection, diagnosis, and early initiation of treatment results in increased rate of infectivity in the society, and it is estimated that untreated smear positive patients can infect on average 10 contacts annually and 20 during the natural history of the disease until death [7].

According to a study conducted in Vietnam, the total median delay was 4 weeks, 3 weeks for patient delay, and health care system was responsible for a delay of 1 week. Nearly 15% of patients with long total delay (≥12 weeks) accounted for 49% of cumulative number of delay weeks [8]. Another study conducted in Tanzania among 639 TB patients showed that patient delay was observed in 35.1% of the patients, with significantly high proportion in females (41%). In addition, diagnosis delay has recorded in 52.9% of the patients, with a high proportion in females (62.1%). In 34.4% of patients, treatment delay was noticed but no significant differences among males and females [9, 10].

Studies conducted at a different setting have showed that different factors contribute for the delay of treatment. For instance, first visit to traditional healers (private clinic), residence, education, severity of illness at first presentation to health facility, patient with good functional status, patients in contact with more than two health providers, not knowing TB symptoms, unemployment, and distance from a health facility were factors associated with TB treatment delays [11–13]. A study conducted in Zambia revealed that, the median diagnostic delay was 8.6 weeks and was significantly associated with sex, education, level of health care encounters, and visiting a private doctor or traditional healer [3]. However, income was found as an independent predictor in a study conducted in Pakistan [4].

Ethiopia has designed various TB diagnosis, treatment, and prevention and control strategies in order to reach at the MDG goals; as a result, it achieved reduction of more than half of most of the MDG targets related to tuberculosis. However, the progress of reduction of the incidence and prevalence has been comparatively slow. Irrespective of all the efforts to achieve early detection of TB and prompt management of cases, significant proportion of TB cases arrived at health facilities after developing serious complications.

In Ethiopia, although there are reports on length of delay to initiate treatment, data regarding proportion of delay among cases and factors associated to delay was scarce. Therefore, this study is aimed at estimating the magnitude of delays in TB treatment and identifying the determinant factors contributing for TB treatment delay.

## 2. Methods and Materials

A facility-based cross-sectional study was conducted from March 1 to April 30, 2018, at Debremarkos town public health institutions. According to the Central statistics report of Ethiopia, Debremarkos town is located in East Gojjam zone, North West Ethiopia, at 300 km far from Addis Ababa, the capital of Ethiopia, and 265 km from Bahirdar, the regional capital. According to the 2015 population projection of major cities in Ethiopia, Debremarkos town has a total population of around 70,000. There are five governmental health institutions (four health centers and one referral hospital) which have been providing Direct Observed Therapy Service (DOTS) to TB patients for the first two months and continuation phase for a varied period of time accordingly. Simple random sampling technique was employed to select Debremarkos town from other seven zonal administrative towns in the region. TB patients who were attending their anti-TB treatment at Debremarkos Health Center, Gozamin Health Center, and Debremarkos Referral Hospital were included in study based on proportional allocation. Systematic random sampling technique was used to draw a sample of 300 TB patients from a total of 1088 patients. Considering list of registered TB patients as a frame, the calculated *K* value was 3. Hence, every three patients attending TB clinics of Debremarkos town were recruited in to the study.

### 2.1. Operational Definitions

*Patient delay* is the time interval between onset of symptoms and the patient's first contact with a healthcare provider [14, 15].

*Diagnostic delay* is the time interval between the first consultations of healthcare provider to diagnosis. Interval more than 3 days was considered as diagnostic delays [10].

*Health care system delay* is the time interval between the dates of visiting health care provider to the initiation of antituberculosis treatment [10, 16].

*Total delay* is time interval from the onset of symptoms to treatment initiation [17].

The patient has *good knowledge about TB*, if the patient can answer correctly the knowledge questions about TB and score equal to and above the median value [11].

The patient has *high perceived stigma about TB*, if the patient scores above and equal to the median value of the questions assessing stigma [11].

### 2.2. Data Analysis

Data was entered using the EPI-info version 7 and then exported to SPSS version 20 for analysis. Binary logistic regression was made to see the crude significant relation of each independent variable with dependent variables. Then, variables with *P* value less than 0.25 in binary logistic regression were taken to multivariable logistic regressions [18] to control the effect of confounding and identify the true relationship between variables. Variables having *P* value of less than 5% with 95% CI were used to declare significant association with the outcome variable. Key descriptive variables were analyzed and presented in texts, tables, figures, and graphs. Ethical clearance letter was obtained from the ethical review committee of the School of Nursing, College of Medicine and Health Science, University of Gondar, with a reference number of C/H/N/U/124/2018. Official letters of permission were taken from zonal health office and respective health institution officers.

The purpose of the study was clearly explained for each study participant, and written verbal consent was taken from participants before interview; besides, they were also informed as participation is on voluntary basis. Patients' name and card number were not taken to assure the privacy and confidentiality of participants.

## 3. Results

In this study, a total of 300 study participants were enrolled from three public health facilities. The median age of the participants was 30 years (IQR 16 years). Of the total 300 study participants, 144 (48%) were males, 118 (39.3%) lived in the rural areas, and 99 (33%) were illiterate. Regarding the occupational status of participants, 48 (16%) were government employed, nearly one-third of study participants (88 (29.3%)) earn a monthly income of less than 500 birr, and a quarter of the participants (76 (25.3%)) have 1500 birr income per month. More than three-fourth of the study participants (266 (82%)) were HIV negative. Just above half of the participants (156 (53%)) were diagnosed as extrapulmonary tuberculosis (EPTB). One hundred twenty (40%) of the participants were living at 6-10 km radius from the nearby health facility (Table 1).

### 3.1. Patient's Clinical Presentation

**Figure 1:**
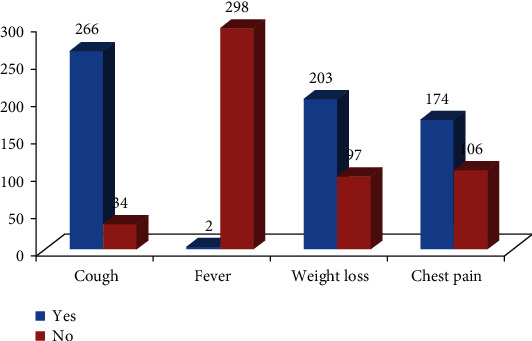
A graph showing clinical presentations of TB patients during onset of illness, Debremarkos Health facilities, Ethiopia.

Closer to half (47%) of the study participants diagnosed pulmonary tuberculosis, and of the total subjects, about 16% were screened HIV positive. Regarding their smoking history, only 6% of participates reported as they have a habit of cigarette smoking. Majority of the study participants (88.7%) revealed as they manifest cough as TB symptom, 203 (67.7%) weight loss, and 174 (64.7%) said they had chest pain (Figure 1).

**Figure 2:**
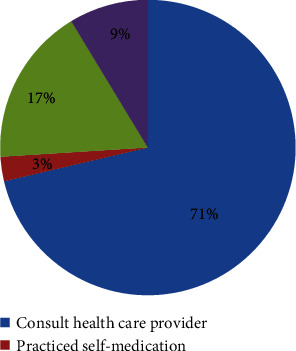
Proportions of patient's first action during the onset of TB symptoms at Debremarkos town public health facilities, North West Ethiopia, 2018.

Although majority of TB (71%) patients had consulted health care provider during the onset of the illness, significant proportion of patients (17.7%) had visit traditional healers as a first priority of care center (Figure 2).

### 3.2. Participant's Knowledge and Perceived Stigma

Almost all the study subjects (99.3%) heard about TB, and more than 92% of the clients are aware about the type of illness they had. About 96% of study subjects know as TB is a contagious illness and curable disease (Table 2). In regard to the source of information, sixty-three percent of participants received information about TB from MOH campaign through media, 16% from formal education, and 12.7% from friends/relatives. The overall knowledge of study subjects showed that above half of the participants had poor knowledge about TB (60%). On the other hand, two-fifth of the participants (40%) perceived that there is high level of stigma for being a TB client. The measure of internal consistency or Cranach's Alpha was 0.744 which showed a better consistency.

#### 3.2.1. Perceived Causes of Delay

Closer to three-fourth of study participants (72.7%) perceived that there is delay for TB treatment. Almost sixty percent of the study subjects explained that their reason of delay to seek medical care was waiting a spontaneous resolution of the symptoms. On the other hand, poor quality of health services delivered in the health facilities account for 13% of study subjects in reason for late presentation to TB treatment.

#### 3.2.2. Proportion of Delay for TB Treatment

About half of the study participants (48%) have shown total delay for TB treatment with a median 23 days (IQR: 19-28 days). The minimum and maximum length of total delays for TB treatment was 13 and 64 days, respectively. Significant proportion of patients (42%) demonstrate patient delay above three weeks of duration with a median value of 20 days (Table 2).

### 3.3. Determinant Factors of Delay in Tuberculosis Treatment

As the multivariable logistic regression result revealed, rural residency has been identified as a determinant variable for delay in TB treatment. Participants who lived in the rural area have 1.14 times higher chance of delay for TB treatment than urban dweller AOR = 1.141 (95% CI: 1.106, 2.608). Educational status is also another variable which showed a significant association with delay for TB treatment; being illiterate patients were two times more likely to delay for TB treatment than participants with an educational status of college and above AOR = 2.35 (95% CI: 1.63, 2.61). Monthly income of participants has found another determinant factor affecting patients delay for TB treatment; as a result, participants who earn below 500 birr per month were six times more likely to delay for TB treatment as compared to those who got 1500 birr and above AOR = 6.37 (95% CI: 1.733, 23.440). Furthermore, participants who visit traditional healers before they came to health facilities were four times more likely to delay for TB treatment AOR = 4.475 (95% CI: 1.204, 16.633). Consulting health care professionals at health facility was found a protective variable for delay for TB treatment AOR = 0.101 (95% CI: 0.020, 0.508) (Table 3).

## 4. Discussion

Early detection of disease and prompt initiation of treatment are essential for an effective TB control program [12]. This study focuses on determining the magnitude of delays to start TB treatment and its contributing factors among TB patients attending DOTS clinics of public health facilities. Patients' delay in seeking care during the onset of TB symptoms and health system delay in diagnosing and initiating antituberculosis treatment contribute for total delays in treatment.

In this study, the median patient delay was 20 days (with IQR: 15-20 days). Forty-two percent (42%) of patients experienced delay greater than 21 days. The median patient delay of this study is shorter than other studies done in Nepal (50 days) [12], Uganda (30 days) [19], southern Ethiopia (30 days) [20], and South-east Ethiopia (63 days) [21]. This might be due to a better health care access to the community and health professionals' campaign in finding cases and promotes early initiation of health services. Moreover, it might be also due to the presence of the urban health extension workers who are responsible to work communicable disease in the community. However, this finding is similar with a study done in Afar region, Ethiopia, which is 20 days [22], and it is also closer to a study done in Malawi that was 14 days (IQR: 15-28 days) [23].

The median health system delay of TB patients in the study area was 4 days (IQR: 3-5 days) which is much lower than previous studies done in Southeast Ethiopia which was 34 days [21], Malawi 59 days (IQR 26-108 days) [23], and Afar, Ethiopia, 33.5 days [22]. This discrepancy might be due to a difference in health professional's ability to detect TB symptoms and availability of advanced diagnostic modality.

The median total delay for treatment among TB patients in the current study was 23 days with an IQR: 19-28 days which is comparatively lower than other previous studies done in Tanzania (136 days) [13], India (55.5 days) [17], and Zimbabwe (36 days) [24]. This wider variation across nations might be due to a difference in the health policy and TB prevention and control treatment strategy of nations. Besides, the finding was also lower than studies conducted in Ethiopia, Southern Ethiopia (45 days) [20], and Afar region (70.5 days) [22], and this could be due to the sociodemography and the infrastructural differences.

Residence of study participants was significantly associated with total delay in TB treatment. Patients who dwell in the rural areas were more likely to delay for TB treatment than the urban inhabitants. This finding is in line with previous studies done in Nigeria [25] and south west Ethiopia [26]. This may be due to transportation problem, lack of money for transportation, poor access health facilities, and lack of health information about TB.

Educational status was also found as another important predicting variable which showed significant association with total delay for TB treatment. The odds of delay among participants who are illiterate, read and write only, and attend primary school only were higher than participants who attended college level and above of education. Hence, being at a higher level of education is a protective variable for delay in TB treatment. This study finding is supported by other studies done in South Africa [9], Dares salaam Tanzania [10], Nigeria [26], and Norway [27]. This could be due to the fact that patients with low educational status would have low level of comprehensive knowledge needed to prevent TB and seek medical care while got infected with TB.

The other determinant variable that showed significant association with total delay for TB treatment was the monthly income of participants. The odds of delay for TB treatment of participants who got less than 500 ETB per month were six times higher than participants who got 1500 ETB and more per month. This finding is in line with other reports done in North West Ethiopia [28]. Patients who did not have adequate income face difficulty to seek health care facilities at the early stage of the disease due to different reasons like fear of medical expenses and transport costs. Hence, they tend to delay for TB treatment.

The odds of delay in TB treatment among those who visited traditional healers before they start ant TB medication were about four times higher than those who contacted health care providers while they manifest symptoms of TB. This finding is concurrent with a report done in southeast Ethiopia [21]. In fact, traditional healers have their own role on providing health services to the community; however, there is longer waiting time to get relief after taking traditional medicines, and most of the healers are neither accredited nor have curable treatment for TB so clients will spend longer time to visit health facilities, and most of them arrive at health facilities with advanced stage of TB.

The types of health facility first consulted by study participants was another variable that showed significantly associated with delay for TB treatment. Study participants who first consulted health post and health center were less likely to delay for TB treatment than those who visited public hospitals initially. However, this finding is in contrast to other study report which was done in Afar region, Ethiopia [22]. This result might be due to the community health network that enhances detection and referral of patients who were suspected to have TB to the appropriate level of diagnosis. On top of this, public hospitals would have high burden of client flow that leads to poor quality of health service and delayed TB diagnosis and initiation of treatment as compared to lower level health facilities; as a result, patients would not prefer to visit hospitals at their first instance of development of TB symptoms.

On the contrary, variables such as age, sex, occupation, type of TB, HIV status, distance to the public health facility, knowledge about TB, and perceived stigma did not show significant association in this study. Similarly, a study conducted in Brazil revealed age and gender had no association with delay in TB treatment [29]; however, studies done in Vietnam [8], south east Ethiopia [11], and Syrian Arab [21] showed a significant association between age and gender.

## 5. Conclusion and Recommendations

In this study, the proportion of delay in tuberculosis treatment was low as compared to other studies but significant numbers of clients delayed to reach at health facilities get appropriate diagnostic service and receive anti-TB medication. Being a rural residence, lower level of education, low economy, visiting traditional healers during the onset of symptoms, and consulting health post and health center other than public hospital were found to be determinant factors of delay for TB treatment. Though the proportion of delay was low as compared to other studies, still, there is a need to strengthen early detection, diagnosis, and treatment strategies on tuberculosis infection to further minimize complications and spread of the disease. Providing regular health education to the community about TB infection emphasizes the rural community and promotes health professionals mass campaign on TB prevention is mandatory. Strengthening health care professional's capacity and technique of early case detection, diagnosis, and treatment; enhancing quality of care in the health facilities; and integrating traditional healers with formal health care system to build a structured referral system of patients are important implications which reduce delays of TB treatment and achieve national TB control program.

## 6. Limitation of the Study

Since information regarding date of onset of tuberculosis symptoms is based on participants' self-report, recall bias may occur. The study does not address some sensitive issues that need to be touched with qualitative design. This study does not include rural health facilities; as a result, its generalizability is in question.

## Figures and Tables

**Table 1:** Sociodemographic characteristics of tuberculosis patients attending DOTS clinic.

Variables	Category	Frequency	Percent
Sex	Male	144	48
	Female	156	52

Age	≤20	24	8
	21-39	187	62.3
	≥40	89	29.7

Religion	Orthodox	284	94.7
	Muslim	13	4.3
	Other catholic	3	1

Residence	Rural	118	39.3
	Urban	182	60.7

Marital status	Unmarried	136	45.3
	Married	164	54.7

Educational status	No education	99	33
	Read and write only	30	10
	Primary school	30	10
	2ndry school and above	141	47

Occupation	Unemployed	56	18.7
	Self-employed	92	30
	Government employed	48	16.0
	Private employed	33	11.0
	Housewife	71	23.3

Income	≤500	88	29.3
	501-1500	136	45.3
	≥1501	76	25.3

Distance from the nearest health center	1-5 km	115	38.3
	6-10 km	120	40.0
	11-20 km	62	20.7
	≥21 km	3	1.0

**Table 2:** The proportion of delays in tuberculosis treatment among DOTS patients at Debremarkos PHF, North West Ethiopia, 2018.

Type of delays	Duration of delays	Gender		Median	IQR	Min	Max
		Male	Female				
Patient delays	<20 days	87	87	20	15-20	12	60
	≥20 days	57	69				
Health system delays	<4 days	99	98	4	3-5	1	9
	≥4 days	45	58				
Total delays	<23 days	76	79	23	19-28	13	64
	≥23 days	68	77				

**Table 3:** The result of logistic regression showing association of variables with total delay for TB treatment at Debremarkos town public health facilities, North West Ethiopia, 2018.

Variables	Categories	Total Rx delay		COR (95% CI)	AOR (95% CI)	*P* value
Age		Yes	No			
	<20 years	10	14	1	1	
	20-39 years	86	101	1.19 (0.5, 2.8)	0.690 (0.2, 2.35)	0.553
	≥40 years	49	40	1.72 (0.69, 4.27)	0.68 (0.16, 2.7)	0.591

Residence	Rural	68	50	1.85 (1.16, 2.96)	1.141 (1.1, 2.61)	0.001^∗^
	Urban	77	105	1	1	

Educational	No education	56	43	1.97 (1.1, 3.7)	2.35 (1.6, 2.6)	0.001^∗∗^
	Read and write	19	11	2.62 (1.1, 6.4)	1.519 (1.3, 7.4)	0.045^∗^
	Primary school	19	11	2.62 (1.1, 6.4)	2.186 (1.5, 9.1)	0.020^∗^
	2ndry school	24	49	0.74 (0.37, 1.48)	0.71 (0.24, 2.1)	0.52
	College+	27	41	1	1	

Income	≤500	44	44	1.62 (0.87, 3.02)	6.37 (1.73, 23.4)	0.005^∗^
	501-1500	72	64	1.82 (1.03, 3.23)	1.44 (0.58, 3.6)	0.440
	≥1501	29	47	1	1	

First actions taken	Consult HCP	113	132	1	1	
	Self-medication	4	4	1.17 (0.29, 4.77)	1.02 (0.21, 5.06)	0.976
	Visit traditional healers	15	6	2.92 (1.09, 7.77)	4.475 (1.2, 16.6)	0.025^∗^
	Use nonprescribed drugs	13	13	1.167 (0.5, 2.63)	1.08 (0.38, 3.04)	0.88

HF first consulted	Health post	5	9	0.327 (0.10, 1.0)	0.101 (0.02, 0.5)	0.005^∗^
	Health center	66	102	0.381 (0.23, 0.6)	0.31 (0.17, 0.57)	0.001^∗∗^
	Public hospital	73	43	1	1	

^∗^Significant association (*P* value less than 0.05); ^∗∗^significant at less than 0.01.

## Data Availability

All the necessary data are included in the manuscript; additional data might be available on request of the corresponding author.
